# Supplementary material for: Co-Inoculation Between Bacteria and Algae from Biological Soil Crusts and Their Effects on the Growth of Poa annua and Sandy Soils Quality
Source: Microorganisms. 2025 Jul 30;13(8):1778. doi: 10.3390/microorganisms13081778 (PMC12388612; doi:10.3390/microorganisms13081778)
Supplement: Supplementary file 1 [file microorganisms-13-01778-s001.zip › microorganisms-3618211-supplementary.pdf]

Co-Inoculation Between Bacteria and Algae from Biological Soil Crusts and Their Effects on the Growth of *Poa annua* and Sandy Soils Quality

Lin Peng [1,†], Xuqiang Xie [1,†], Man Chen [1], Fengjie Qiao [1], Xingyu Liu [1], Yutong Zhao [1], Xiawei Peng [1,*] and Fangchun Liu [2,*]

[1]School of Biological Sciences and Technology, Beijing Forestry University, Beijing 100083, China

[2]Shandong Academy of Forestry, Jinan 250014, China

*Correspondence address: School of Biological Sciences and Technology, Beijing Forestry University, 35 Qinghua East Road, Beijing 100083, China

Corresponding authors: Xiawei Peng, xiaweipeng@163.com； Fangchun Liu, fchliu@126.com

Supplementary Figures

The biological crusts were collected from the karst desertification area in Yunnan Province. This area has no obvious ground vegetation, with an altitude of 1500 meters, and belongs to a typical South Asian tropical monsoon climate region. This study selected areas with less external interference, similar terrain features, and higher stability of the biological crusts as the research objects. Following the principle of random distribution, 5 sampling points were arranged, with a spacing of more than 2 meters between each point. Using the random sampling method, repeated sampling was conducted on different types of biological crust samples, mainly including lithogenic and soil-forming biological crusts.

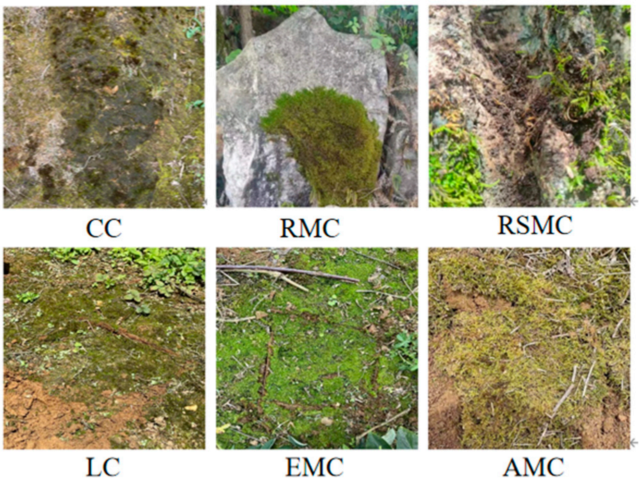

Figure S1. Biological soil crusts at different developmental stages. CC: algae crust; RMC: lithophytic moss crust; RSMC: rocky soil mixed moss crust; LC: Lichen crust; EMC: Early moss crust; AMC: Advanced lichen crust.

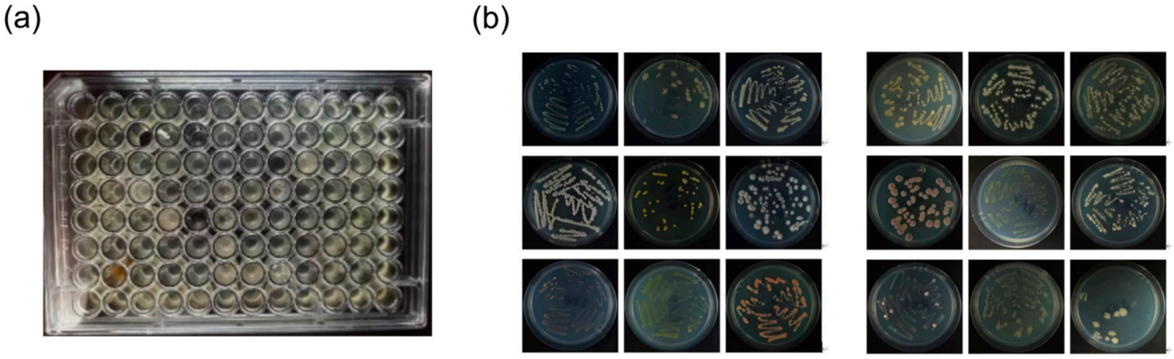

Figure S2. Separation and purification of bacteria. (a) Bacteria cultured in 96-well plates for two weeks. (b) Culture morphology of some strains on plates.

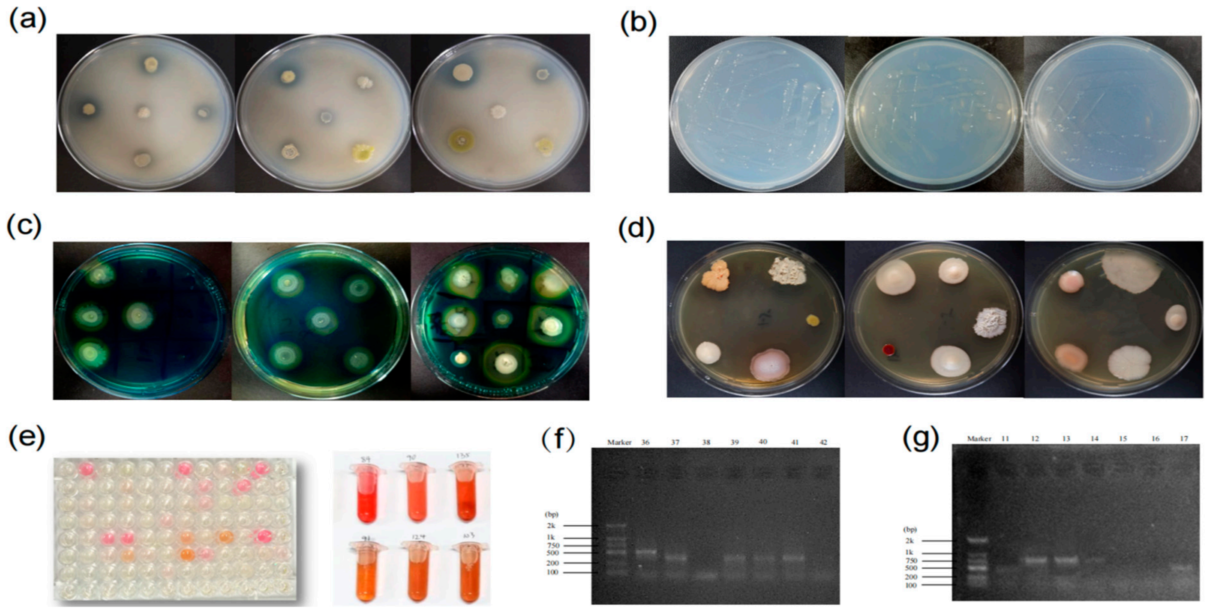

Figure S3. Determination of functional indexes of bacteria. (a) Screening of phosphorus solubilizing strains; (b) Screening of potassium solubilizing strains; (c) Screening of siderophore strains; (d) Screening of EPS producing strains; (e) Screening of IAA-producing strains; (f) Screening of nitrogen-fixing strains; (g) Screening of ACC deaminase producing strains.

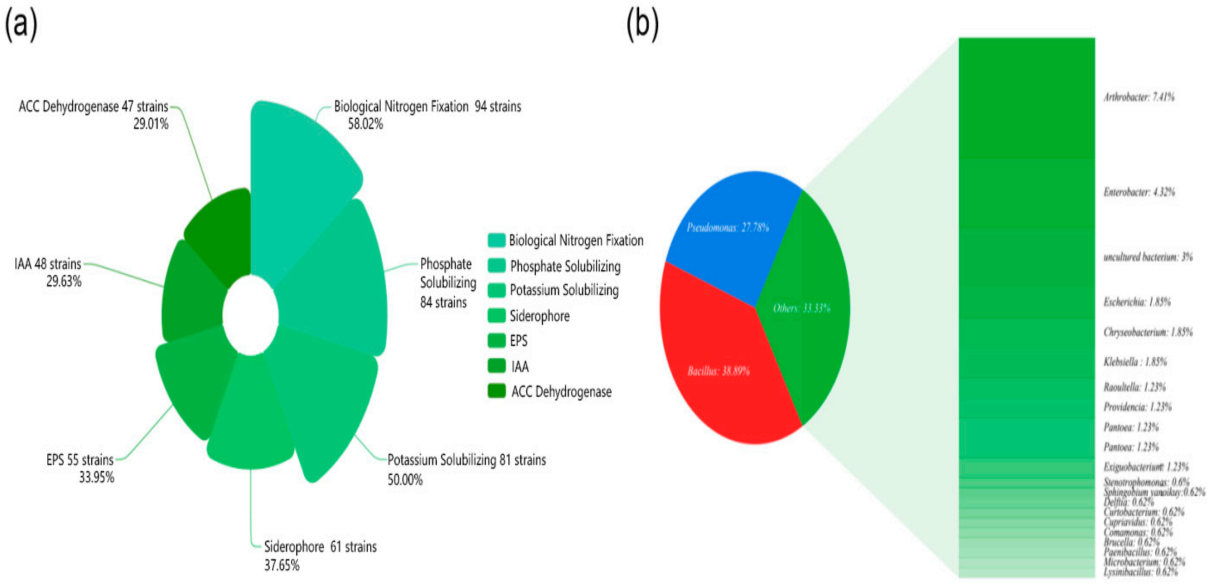

Figure S4. The proportion of functional bacteria (a) and results of identification (b) of 162 strains.

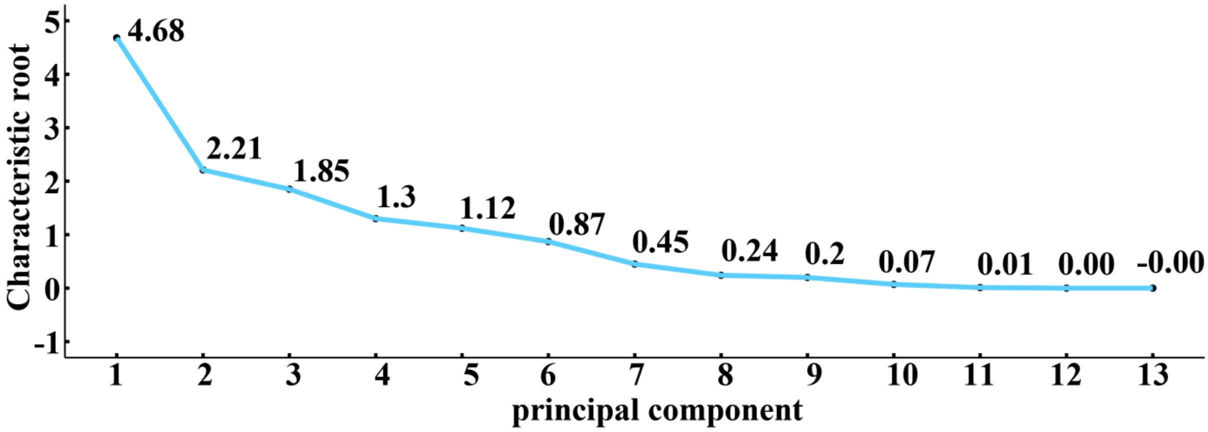

Figure S5. Scree plot of PCA.

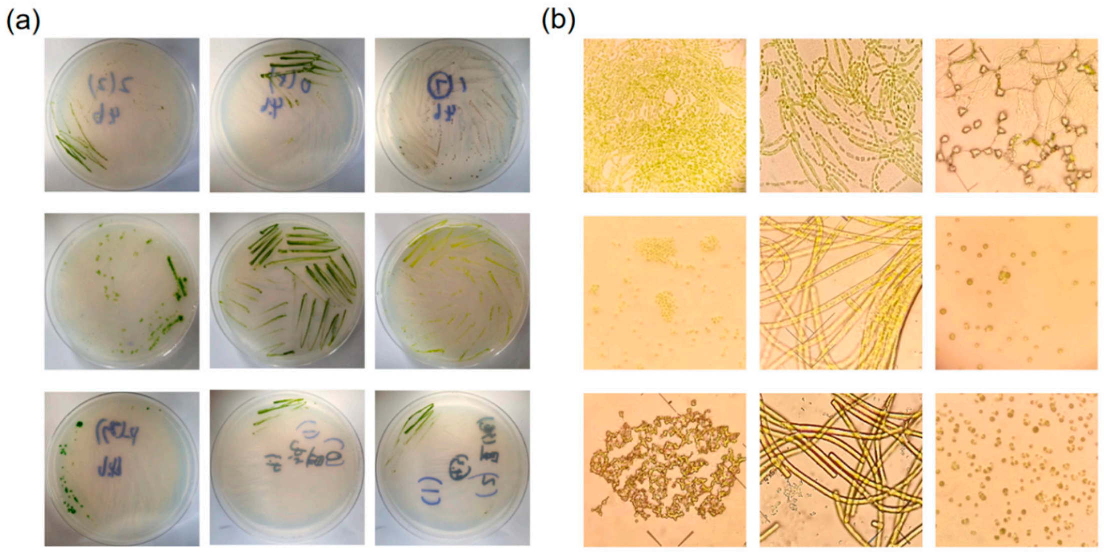

Figure S6. Separation and purification of algae. (a) Growth morphology of some algal strains on the plate. (b) Microscopic morphology of some algal strains under microscope. Note: The above microscopic images were taken under a 40 (objective) ×10 (eyepiece) power microscope.

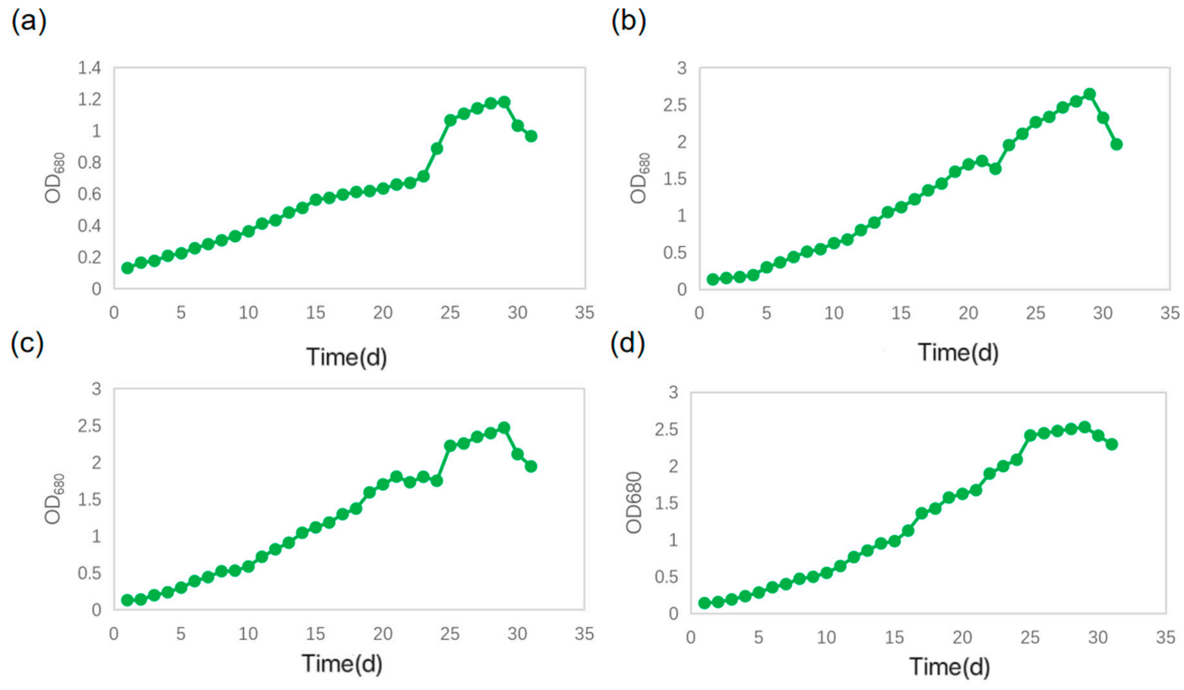

Figure S7. Comparison of growth rates of （a）CC-1、（b）LC-8、（c）EMC-7 and（d）AMC-5.

Supplementary Tables

Table S1. The comparison results of 16S gene sequence of 36 strains in EzBioCloud database

**Number**	**The closest related species**	**Homologous value**
CC-4 26	Pseudomonas neuropathica P155	99.19%
CC-4 6	Pseudomonas pharynges BML-PP036	99.21%
CC-5 22	Paenibacillus mucilaginosus VKPM B-7519	99.65%
LC-2 5	Cupriavidus basilensis DSM 11853	99.02%
LC-2 7	*MDDN_s* SR-86	99.04%
LC-4 7	*WJYD_s* RIT712	99.38%
LC-4 18	Bacillus velezensis CR-502	99.86%
EMC-3 24	Pseudomonas pharynges BML-PP036	99.64%
EMC-4 11	Bacillus velezensis CR-502	99.64%
EMC-4 28	Bacillus zanthoxyli 1433	99.72%
AMC-2 28	Delftia acidovorans 2167	99.31%
AMC-3 22	Exiguobacterium acetylicum DSM 20416	99.18%
AMC-4 10	Pseudomonas hunanensis LV	99.72%
AMC-4 34	Bacillus velezensis CR-502	99.64%
JZ	Paenibacillus mucilaginosus VKPM B-7519	99.72%
L1 28	Pseudomonas pharynges BML-PP036	99.78%
L1 8	Bacillus paranthracis Mn5	99.38%
L2 8	Bacillus arachidis SY8	99.57%
L2 9	Pseudomonas pharynges BML-PP036	99.64%
L3 31	Acinetobacter calcoaceticus DSM 30006	99.44%
L3 9	Bacillus paramobilis BML-BC017	99.45%
L5 19	Sphingobium yanoikuyae ATCC 51230	99.42%
L5 30	Acinetobacter pittii CIP 70.29	99.93%
LS 176	Curtobacterium albidum DSM 20512	99.21%
LS 238	Bacillus velezensis CR-502	99.29%
MGS 25	Acinetobacter oleivorans DR1	99.24%
MGS 1	Pantoea brenneri LMG 5343	99.93%
RMC1 20	Bacillus velezensis CR-502	99.86%
RMC1 27	Stenotrophomonas indicatrix WS40	99.31%
RMC2 16	Bacillus zanthoxyli 1433	99.51%
RMC4 3	Arthrobacter bambusae GM18	98.50%
RSMC1 10	Bacillus arachidis SY8	99.43%
RSMC1 21	Bacillus velezensis CR-502	99.43%
RSMC2 27	Arthrobacter ipis IA7	99.09%
RSMC3 4	Pseudomonas pharynges BML-PP036	99.21%
RSMC4 23	Pseudomonas simiae OLi	99.55%

Table S2. Quantitative results of functional bacteria in 36 strains

Number	Phosphorus soluble content (mg/L)	Nitrogenase activity (ng/L)	Potassium content (mg/L)	Ferritic activity SU(%)	IAA level (mg/L)	EPS level (mg/L)	ACC activity （U/mg）
CC-4 26	38.09	152.07	1.68	—	—	—	—
CC-4 6	54.86	83.5	—	—	9.91	—	—
CC-5 22	—	—	2.97	—	—	15.39	0.1248
LC-2 5	—	242.64	—	—	8.68	—	—
LC-2 7	—	—	1.94	64.2	9.78	—	0.5735
LC-4 1	50.26	—	2.68	—	—	9.59	—
LC-4 18	—	—	1.94	—	—	22.32	—
EMC-324	28.36	—	2.66	—	—	10.22	0.2431
EMC-411	36.69	72.29	—	—	—	—	—
EMC-428	34.64	124.93	—	—	23.93	—	—
AMC-322	—	—	—	—	8.71	—	—
AMC-410	—	87.93	—	75.4	—	—	0.2322
AMC-428	—	104.14	2.35	—	—	—	—
AMC-434	23.05	—	2.15	—	9.32	—	0.3354
JZ	50.31	140.93	3.24	—	25.16	23.88	—
L1 28	—	174.57	1.61	—	—	—	—
L1 8	49.96	—	—	—	—	—	0.4324
L2 8	38.86	161.07	1.53	77.8	8.4	—	—
L2 9	86.7	120.86	1.91	54.6	—	—	0.2377
L3 31	—	93.07	1.79	70.4	8.5	26.38	—
L3 9	30.35	—	—	—	—	9.67	0.3672
L5 19	31.96	90.07	—	—	—	—	—
LS 238	34.45	132.29	—	—	31.97	—	—
MGS 25	76.74	143.21	2.45	80.4	23.83	—	0.3842
MGS1	—	—	2.35	—	12.74	—	0.1037
RMC 120	33.54	158.71	1.24	—	—	—	—
RMC 127	47.37	149.21	—	—	—	—	—
RMC 216	—	116.57	—	—	—	—	—
RMC43	—	—	—	63.7	—	—	—
RSMC 110	—	156.36	2.7	—	—	—	0.0924
RSMC1	—	140.64	3.03	—	—	—	—

21							
RSMC2<br>27	20.96	—	—	—	12.92	14.79	—
RSMC3<br>4	30.25	—	—	—	—	10.21	—
RSMC4<br>23	51.38	—	—	70.3	—	—	—

Note: "—" indicates that the strain does not have this function

Table S3. Growth indexes of *Poa annua* under different treatments

Number	Total Length (cm)	Plant Height (cm)	Root Length (cm)	Total Weight (g)	Above-ground Biomass (g)	Under-ground Biomass (g)
S	7.5±0.8k	3.9±0.3j	3.6±0.7i	0.40±0.01j	0.17±0.02l	0.23±0.04g
PYJ	8.2±0.6k	4.5±0.9ij	3.7±0.9i	0.40±0.08j	0.19±0.01j	0.21±0.07i
LS 176	15.9±0.8cdefg	7.4±0.4de	8.5±0.4cde	1.29±0.01c	0.54±0.01b	0.75±0.01d
LS 238	16.5±0.8bcde	10.4±0.5b	6.1±0.3h	1.33±0.06c	0.54±0.03b	0.79±0.04d
JZ	16.3±1.5bcdef	7.5±1.2d	8.8±1.3bc	1.05±0.10de	0.42±0.08de	0.64±0.04e
RSMC4 23	19.6±1a	13.5±0.7a	6.1±1.3h	1.28±0.06c	0.51±0.02c	0.78±0.04d
RSMC3 4	17.4±0.9b	7.7±0.4cd	9.7±0.5a	2.02±0.11a	0.64±0.03a	1.39±0.07a
RSMC2 27	15.8±0.8cdefgh	7.2±0.4def	8.6±0.4cd	1.62±0.08b	0.65±0.03a	0.96±0.05c
RMC4 3	16.2±0.8cdef	10.2±0.5b	6±0.3h	1.62±0.05e	0.64±0.00a	0.98±0.05c
RMC1 20	12.1±1.6j	4.8±1.2i	7.3±0.4g	0.87±0.04f	0.39±0.02ef	0.48±0.02f
MGS 25	15.4±0.8defgh	7.5±0.4d	7.9±0.4efg	1.13±0.06d	0.48±0.06c	0.64±0.03e
L2 9	13.9±0.7i	5.6±0.3h	8.3±0.4cde	0.69±0.03hi	0.21±0.01j	0.48±0.02f
L1 28	14.8±0.7ghi	6.7±0.3fg	8.1±1.4def	1.24±0.01k	0.51±0.01b	0.72±0.01d
AMC-4 34	16.8±1.8bc	7.5±0.4d	9.3±0.5ab	0.77±0.04gh	0.30±0.01h	0.47±0.02f
AMC-4 10	15.3±0.8efgh	6.8±0.3efg	8.5±1.4cde	1.30±0.06c	0.24±0.01i	1.05±0.05b
EMC-4 28	16.6±0.8bcd	7.3±0.4de	9.3±0.5ab	1.07±0.05de	0.43±0.02d	0.64±0.03e
LC-4 7	15.7±1.8cdefgh	8.2±0.4c	7.5±0.4fg	1.3336±0.0651c	0.33±0.07g	1.00±0.04c
LC-4 1	14.6±0.7hi	6.4±0.3g	8.2±0.4cde	0.6357±0.031i	0.25±0.01i	0.37±0.02g

LC-4 18	15.2±0.8fgh	7.8±0.4cd	7.4±0.4g	0.634±0.031i	0.36±0.05f	0.26±0.01h
CC-4 26	16.2±0.8cdef	6.5±0.3g	9.7±0.5a	0.81±0.04fg	0.29±0.01h	0.51±0.02f

Note: The statistical differences within a column are indicated by different letters (one-way ANOVA, α = 0.05).

Table S4. Physical and chemical properties under different treatments

Number	pH	EC(μS/cm)	EPS(mg/g)	AN(mg/kg)	AP(mg/kg)	AK(mg/kg)
S	7.24±0.05cdef	108.53±3.37h	0.1±0.01k	51.33±14.57g	2.28±0.05g	33.2±0.75f
PYJ	7.22±0.09cdef	130.43±3.56gh	0.12±0k	100.33±21.39def	3.1±0.07fg	38.33±0.5e
LS 176	7.43±0.03b	170.4±11.14abcde	0.35±0.02h	126±14cde	3.63±0.5efg	46.67±1.1bc
LS 238	7.05±0.03h	172.7±6.02abcde	0.3±0.01j	77±14fg	2.96±0.03fg	34.33±1.87f
JZ	7.16±0.13defgh	169.9±5.69abcde	0.44±0.02ab	163.33±14.57bc	7.64±0.83a	44.73±1.91c
RSMC4 23	7.17±0.03defgh	193.2±9.8a	0.45±0.01a	93.33±24.58efg	6.53±0.31abc	32.83±1.83f
RSMC3 4	7.33±0.05bc	188.27±18.96a	0.38±0.02ef	100.33±41.02def	4.09±0.09def	38.53±0.65e
RSMC2 27	7.2±0.04def	154.67±21.82defg	0.41±0.01de	109.67±4.04def	7.03±1.56ab	32.83±1.36f
RMC4 3	7.15±0.03efgh	160.77±30.55bcdef	0.41±0.01cd	291.67±35.5a	5.75±0.46bcd	51.73±0.86a
RMC1 20	7.24±0.03cdef	137.57±16.55fg	0.38±0.01fg	107.33±33.08def	7.77±1.62a	33.17±0.83f
MGS 25	7.16±0.03efgh	178.13±7.94abcd	0.36±0.03h	144.67±10.69cd	7.8±0.79a	47.73±0.57b
L2 9	7.29±0.02cd	161.03±4.66bcdef	0.29±0.01j	144.67±10.69cd	4.94±0.49cde	38.1±0.7e
L1 28	7.24±0.06cde	156.7±17.15cdef	0.43±0bcd	128.33±14.57cde	6.52±1.13abc	47.63±0.93b
AMC-4 34	7.71±0.06a	152.5±27.71efg	0.3±0.01j	114.33±14.57def	6.53±0.95abc	26.37±2.57h
AMC-4 10	7.11±0.02fgh	177.9±8.75abcd	0.36±0.02fgh	121.33±24.58cdef	6.57±0.27abc	44.5±1.84c
EMC-4 28	7.21±0.07cdef	171.3±6.27abcde	0.43±0.01abc	98±14ef	6.26±1.24abc	41.07±0.64d
LC-4 7	7.33±0.02bc	177.33±16.75abcde	0.37±0.02fgh	126±32.08cde	4.3±0.5def	47.4±1.3b
LC-4 1	7.18±0.04defg	181.8±7.54abc	0.33±0.01i	144.67±14.57cd	7.44±1.14ab	30.57±1.84g
LC-4 18	7.13±0.05efgh	184.97±23.61ab	0.29±0.02j	88.67±4.04efg	6.12±1.12abc	41.07±1.11d
CC-4 26	7.06±0.05gh	168.4±13.91abcde	0.36±0.02gh	203±38.97b	4.14±0.57def	39.67±0.81de

Note: The statistical differences within a column are indicated by different letters (one-way ANOVA, α = 0.05).

Table S5. Mechanical stability of aggregates under different treatments

Number	MWD(mm)	GMD(mm)
S	1.15±0.02fg	0.89±0.02ef
PYJ	1.1±0.02g	0.85±0.03f
LS 176	1.16±0.01defg	0.93±0.01def
LS 238	1.35±0.04abc	1.12±0.03abc
JZ	1.3±0.02bcde	1.06±0.03bcd
RSMC4 23	1.16±0.24defg	0.92±0.18def
RSMC3 4	1.11±0.02g	0.89±0.02ef
RSMC2 27	1.21±0cdefg	0.97±0def
RMC4 3	1.24±0.06cdefg	0.98±0.06cdef
RMC1 20	1.21±0.01cdefg	0.98±0cdef
MGS 25	1.23±0.03cdefg	1±0.03cde
L2 9	1.15±0.03efg	0.92±0.03def
L1 28	1.22±0cdefg	0.97±0.01def
AMC-4 34	1.18±0.02defg	0.91±0.07ef
AMC-4 10	1.28±0.16bcdef	1.01±0.19bcde
EMC-4 28	1.31±0.01bcd	1.06±0bcd
LC-4 7	1.46±0.1a	1.24±0.1a
LC-4 1	1.12±0.02g	0.89±0ef
LC-4 18	1.4±0.03ab	1.15±0.01ab
CC-4 26	1.19±0.03defg	0.94±0.02def

Note: The statistical differences within a column are indicated by different letters (one-way ANOVA, $\alpha = 0.05$).

MWD: Mean Weight Diameter, GMD: Geometric Mean Diameter.

Table S6. KMO and Bartlett's test

Test Method		Test Value
KMO Test		0.841
**Bartlett's Test of Sphericity**	**Approximate Chi-squared Value**	1114.055
	**Degree of Freedom**	78
	**P-Value**	0

Table S7. Total variance explained of the first five principal components

Principal Component	Eigenvalue	Variance Explained（%）	Cummulative（%）
1	4.678	35.986	35.986
2	2.207	16.981	52.966
3	1.854	14.261	67.228
4	1.305	10.037	77.265
5	1.12	8.618	85.883

Table S8. Ranking of principal components of strains

Number	Principal Component 1	Principal Component 2	Principal Component 3	Principal Component 4	Principal Component 5	Total	Ranking
JZ	1.81	1.19	0.21	0.47	0.73	1.16	1
EMC-4 28	1.65	0	-0.42	0.83	0.83	0.8	2
MGS 25	1.07	0.42	0.31	-0.1	0.77	0.65	3
RMC4 3	1.04	2.67	0.37	-3.61	0.18	0.62	4
LC-4 7	2.2	1.27	-3.18	0	-0.43	0.6	5
RSMC4 23	2.24	-1.84	0.74	-0.85	-0.15	0.58	6
RSMC2 27	1.97	-2.44	0.43	-0.16	1.12	0.51	7
AMC-4 10	1.12	0.56	-0.18	-0.01	-0.41	0.51	8
L1 28	-0.96	2.41	0.85	0.97	0.42	0.37	9
RSMC3 4	2.34	-2.77	0.91	-1.17	-1.28	0.32	10
CC-4 26	-0.13	1.32	1.41	0.3	-1.58	0.31	11
LC-4 18	0.18	0.82	-2.1	1.59	0.75	0.15	12
AMC-4 34	-0.45	-0.52	1.73	1.84	-1.23	0.09	13
LC-4 1	-1.16	-0.36	1.93	0.61	0.6	-0.11	14
RMC1 20	-0.63	-1.28	0.13	0.24	2.74	-0.19	15
LS 238	1.26	-1.18	-2.51	0.41	-1.69	-0.24	16
LS 176	-1.43	1.31	0.58	-0.19	0.05	-0.26	17
L2 9	-1.56	0.65	1.12	0.61	-1.28	-0.4	18
PYJ	-5.04	-0.83	-0.65	-1.38	0.22	-2.52	19
S	-5.52	-1.39	-1.66	-0.4	-0.38	-2.95	20

Table S9. Blast comparison results of tufA gene sequences of four algae

Number	The closest related species	Homologous value
CC-1	Chlorella protothecoides KY613608.1	98.04%
LC-8	Coelastrella thermophila MH176136.1	96.54%
EMC-7	Scenedesmus vacuolatus HG514403.1	98.02%
AMC-5	Scenedesmus vacuolatus HG514403.1	97.97%

Supplementary Information

Note S1. Isolation of Functional bacteria

First, 1 g of crust soil was added to 99 mL of sterilized distilled water, followed by shaking the mixture for 30 minutes. Subsequently, the mixture was gradually diluted, and the optimal dilution degree was determined by the high-throughput bacterial screening method (Zhang et al.2021). The diluted bacterial solution was placed in a 96-well plate and cultured in the dark at room temperature for 14 days. Subsequently, the sterilized toothpicks were used to dip into the turbid wells and inoculated onto 10% TSB solid medium. After the growth of single colonies, the samples were transferred to 10% TSB liquid medium and cultured in a shaking incubator (30 °C, 180 rpm) for 24 to 48 hours, after which 862 strains were screened (Supporting Information: Figure S2). Whereafter, 162 strains were further selected for by the functional screening for further studies (Supporting Information: Figure S3、S4), in which the functional screening was composed of inorganic phosphorus removal function (Sharma et al. 2013), potassium removal function (Etesami et al. 2017), nitrogen fixation function (de Bruijn, 2015), iron-producing carrier function (Ahmed, E. and Holmström, S.J.M, 2014), IAA production function (Patten et al. 1996), EPS production function (Decho & Gutierrez, 2017) and ACC deaminase production function (Bernard et al. 1998). Subsequently, the pathogenic genera could be excluded based on the data of the beneficial bacteria genera in previous studies, and a certain degree of classification redundancy could be avoided (Pathogen-Host Interactions Database(PHI-base). http://www.phi-base.org). Finally, 36 strains of bacteria were obtained (Supporting Information: Table S1、S2), and these strains were inoculated into 10% TSB liquid medium and cultured in a shaking incubator (30 °C, 180rpm) for 36 to 48 hours, followed by adjusting the mixture to a concentration of 108 CFU/mL with sterilized distilled water. Whereafter, the bacterial suspension and Poa annua were cultivated together for 45 days. Meanwhile, set water (S) and liquid culture medium (PYJ) were applied for the control groups. The 12 measured indicators (Poa annua growth index and soil physicochemical property indicators) were subjected to principal component analysis, while the 5 principal components (with characteristic roots greater than 1) were selected for scoring and ranking, and the top 4 strains were ultimately obtained (Supporting Information: Table S3-S8、Figure S5).

Note S2. Isolation of Algae

In this section, 1 g of crust soil was added to 30 mL of BG11 liquid medium, followed by shaking the mixture in a shaker (30 °C, 180 rpm), and the samples were then placed under 2700 lx of light at 27 ℃ for 2 weeks. When the BG11 liquid medium turned green, a sterilized toothpick would be employed to stir thoroughly in the medium, followed by transferring it to the BG11 solid medium. The purification process was repeated for cultivation until the algal strain exhibited a consistent morphology without bacterial contamination. Moreover, an optical microscope was employed to confirm the sample as a pure algal strain. Based on the above procedures, a total of 33 strains were obtained (Supporting Information: Figure S6), and these pure algal strains were simultaneously inoculated onto the BG11 solid medium, followed by the observation and comparison of their growth rates on the plate. Whereafter, four faster-growing algal strains were selected. Additionally, a single

algal colony was picked and inoculated onto 30 mL of BG11 liquid medium, followed by cultivating it with 2700 lx of light at 27 ℃. Moreover, the OD680 value was measured every 24 hours, and the growth curve was drawn (Lu Ninghai 2004) (Supporting Information: Figure S7).

Note S3. Soil physiochemical and functional measurements

Soil pH was determined from 2.5 g fresh soil sample mixed with 12.5 ml of Milli-Q water and shaken for 1 h at 180 rpm and allowed to settle before reading with a Delta pH-meter (Mettler-Toledo Instruments, Columbus, OH, USA). Soil alkali-hydrolyzable nitrogen is determined by the alkali-hydrolysis diffusion method (Lu Rukun. Soil Agricultural Chemistry Analysis Methods [M]. Beijing: China Agricultural Science and Technology Press, 2000.04.). The available phosphorus was extracted with sodium bicarbonate and then determined by the molybdenum-silver antimony colorimetric method (Olsen method) using a spectrophotometer (UV-1800, Shimadzu, Japan). Soil total nitrogen (TN) and total phosphorus (TP) contents were determined by elemental analyzer (EA3000, EuroVector, Italy). The available potassium in the soil was determined by the flame emission spectrophotometer (FP6450, INESA, China). Soil polysaccharides were determined by Anthrone-Sulfuric Acid Method: Add 5.0 g of dried soil (filtered through a 0.25 mm sieve) to 25 mL of 0.5 mol/L H2SO4. Incubate at 80°C in a water bath at 150 rpm for 1 h, then centrifuge at 4000 rpm for 10 min, and collect the supernatant. Repeat the extraction once and combine the supernatants. Dilute to 50 mL and measure the concentration using a spectrophotometer (UV-1800, Shimadzu, Japan). The structure and stability of soil aggregates were determined by the aggregate analyzer (JXSF-U2-WT,Jing Xin, China): Allow the fresh soil sample (avoid squeezing) to naturally air-dry. Filter it through a 2 mm sieve and weigh 50 g (accurate to 0.01 g) and place it in an aluminum box. Record the weight of the dry soil (W_0). Transfer the pre-wetted soil sample to the top 5 mm sieve. Immerse it in water at room temperature or at a constant temperature of 20°C and oscillate it up and down at a frequency of 30 times per minute (amplitude 3 cm) for 30 minutes. Collect the aggregates in each sieve layer (5 mm, 2 mm, 1 mm, 0.5 mm, 0.25 mm) and the bottom plate (<0.25 mm) step by step. Finally, transfer each level of aggregates to the aluminum box, dry them at 105°C until constant weight, and weigh them (W_1, W_2, ..., W_5). Finally, calculate their structure and stability.

Overall, these soil nutrient pools and processes constitute a good proxy of nutrient cycling, organic matter decomposition, biological productivity, and build-up of nutrient pools (Bell et al. 2013; Bradford et al. 2014; Jing et al. 2015; Maestre et al. 2012).

Note S4. Microbial biomass measurements

The soil microbial biomass carbon (MBC) and microbial biomass nitrogen (MBN) were determined by chloroform-fumigation-extraction method (Brookes et al. 1985 and Vance et al. 1987).

References:

Zhang, J.-Y.; Liu, Y.-X.; Guo, X.-X.; Qin, Y.; Ruben, G.-O.; Lefert, P.-S.&Bai, Y. High-throughput cultivation and identification of bacteria from the plant root microbiota. *Nature Protocols*, 2021, 16(2): 988-1012.

Lu N.-h. Application of Algal Cell Suspension Culture in High-throughput Biological Screening for Antibacterial and Insec-ticidal Agents. Lanzhou: Gansu Agricultural University, 2004.

Sharma, S.-B.; Sayyed, R.-Z.; Trivedi, M.-H.; Gobi, T.-A. Phosphate solubilizing microbes: sustainable approach for managing phosphorus deficiency in agricultural soils. *SpringerPlus*, 2013, 2, 587. https://doi.org/10.1186/2193-1801-2-587

Etesami H.; Emami S.; Alikhani H.-A.; Potassium solubilizing bacteria(KSB): mechanisms, promotion of plant growth, and future prospects-a review, J. Soil Sci. *Plant Nutr.,* 2017, 17:4, 897–911.

de Bruijn, F. Biological Nitrogen Fixation. In: Lugtenberg, B. (eds) Principles of Plant-Microbe Interactions. *Springer, Cham*, 2015, https://doi.org/10.1007/978-3-319-08575-3_23

Ahmed, E. and Holmström, S.J.M. Siderophores in environmental research. *Microbial Biotechnology*, 2014, 7: 196-208. https://doi.org/10.1111/1751-7915.12117

Patten C.-L.; Glick B.-R. Bacterial biosynthesis of indole-3-acetic acid. *Can J Microbiol.* 1996, 42(3):207-20. doi: 10.1139/m96-032. PMID: 8868227.

Decho, A.-W. & Gutierrez, T. Microbial extracellular polymeric substances (EPS) in ocean systems. *Frontiers in Microbiology*, 2017, 8, Article 922. https://doi.org/10.3389/fmicb.2017.00922

Bernard R.; Glick, D.-M.; Penrose, Li J.-P. A Model For the Lowering of Plant Ethylene Concentrations by Plant Growth-promoting Bacteria. *Journal of Theoretical Biology*, 1998, 190(1), 63-68. https://doi.org/10.1006/jtbi.1997.0532.

Bell, C.-W.; Fricks, B.-E.; Rocca, J.-D.; Steinweg, J.-M.; McMahon, S.-K.; Wallenstein, M.-D. High-throughput fluorometric measurement of potential soil extracellular enzyme activities. *J Vis Exp*, 2013, e50961.

Brookes, P.-C.; Landman, A.; Pruden, G.; Jenkinson, D.-S.; Chloroform fumigation and the release of soil nitrogen: A rapid direct extraction method to measure microbial biomass nitrogen in soil. *Soil Biol. Biochem.* 1985, 17, 837–842. https://doi.org/10.1016/0038-0717(85)90144-0.

Vance, E.-D.; Brookes, P.-C.; Jenkinson, D.-S.; An extraction method for measuring soil microbial biomass C. *Soil Biol. Biochem.* 1987, 19, 703–707. https://doi.org/10.1016/ 0038-0717(87)90052-6.

Lu R.-K. Soil Agricultural Chemistry Analysis Methods [M]. Beijing: China Agricultural Science and Technology Press, 2000.04.
